# 18F-FDG brain/cerebellum-to-liver ratios as prognostic factors

**DOI:** 10.1038/s41408-025-01357-y

**Published:** 2025-08-27

**Authors:** David Morland, Eric Durot

**Affiliations:** 1 Médecine Nucléaire, Institut Godinot, Reims, France; 2 CReSTIC, UR 3804, Université de Reims Champagne-Ardenne, Reims, France; 3 Laboratoire de Biophysique, UFR de Médecine, Université de Reims Champagne-Ardenne, Reims, France; 4 Hématologie Clinique, CHU de Reims, Reims, France

**Keywords:** Risk factors, Cancer imaging

## To the Editor:

We read with great interest the article by Dingli et al. [1] in which they study the ratio of brain to liver 18F-FDG uptake in a cohort of patients with multiple myeloma who had PET/CT imaging performed immediately prior to chimeric antigen receptor T cell therapy. Based on this index, the authors were able to separate two groups with different prognoses: a group with a ratio <2.5, with an inferior survival, and a group with a ratio >2.5, with a better survival.

The idea that tumor mass may compete with the brain for glucose uptake is not new and was notably demonstrated by Hanaoka et al. [2] in 2010 in non-Hodkin lymphomas. On this basis, our team introduced in 2021 a cerebellum/liver ratio (CLIP: Cerebellum/Liver Index for Prognosis) as a prognostic factor for lymphomas [3]. The choice of the cerebellum rather than the whole brain was preferred for two reasons: the brain is not systematically included in its entirety in the field of view of PET/CT, and the cerebellum in adults is less subject to metabolic changes linked to possible neurodegenerative pathologies. We also preferred to use the SUVmax of the cerebellum rather than an average, to enhance the reproducibility of the measurement [3, 4]. As in the article by Dingli et al. [1], we used the mean liver uptake to normalize cerebellar uptake. Although tumor mass may also compete with liver uptake, we hypothesized that this effect would be less sustained than in the cerebellum/brain, which is an almost exclusive consumer of glucose, unlike the liver.

CLIP has been studied by our team in three main types of lymphoid malignancies: follicular lymphomas [4], diffuse large-cell B lymphomas [3] and Post-Transplant Lymphoproliferative Disorders (PTLD) [5]. These studies point to a negative impact of low CLIP on progression-free survival (PFS) with hazard ratio ranging from 3.1 to 3.7, and overall survival [5]. A summary can be found in Table 1. The link between total metabolic tumor volume (TMTV) and cerebellar and hepatic uptake was also briefly discussed in a paper on Hodgkin’s lymphoma in a retrospective cohort of 163 patients [6].

**Table 1:** Summary of studies testing Cerebellum-to-Liver ratio for Progression-Free Survival prediction (PFS).

	Follicular lymphomas [4]	Diffuse large B-cell lymphomas [3]	Post-transplant lymphoproliferative disorders [5]
Number of patients	46	95	97
Optimal threshold	4.0	3.24	2.605
	5-year PFS	5-year PFS	
Survival data	78.6% (high CLIP)	68.0% (high CLIP)	NA
	42.0% (low CLIP)	32.0% (low CLIP)	
Hazard Ratio when low CLIP (univariate)	3.1 (1.01–9.4) *p* = 0.049*	3.4 (1.8–6.7) *p* < 0.001*	3.7 (1.7–7.8) *p* < 0.001*
Hazard Ratio when low CLIP (multivariate)	2.8 (0.9–8.8) *p* = 0.07	2.1 (1.0–4.5) *p* = 0.04*	4.2 (1.9–9.4) *p* < 0.001*

*NA* not available.

^*^p < 0.05.

All sources studying a cerebrum/cerebellum to liver ratio, including the article by Dingli et al. [1] converge on an optimal threshold of between 2 and 4, depending on the publication. Dichotomization of continuous variables has, however, many drawbacks including the loss of information, the risk of misclassification and the problem of comparability of the results. In the study by Murairi et al. [7], CLIP was studied in particular on the cohort of the “Real Time Tailored Therapy” (RT3) study which is an observational multi-center study that aimed to report the histological result and molecular characterization of the patient tumor material with previously untreated diffuse large B-cell lymphoma. This study confirms the association of CLIP with 3-year PFS and demonstrates that the use of spline modeling of the continuous variable may be more interesting than a cut-off approach. In particular, it allows us to consider that higher values of CLIP have less impact on PFS than lower values.

The use of an 18F-FDG ratio based on cerebral/cerebellar uptake is therefore promising. However, certain confounding factors should not be overlooked: age may moderately influence cerebral fixation, notably via neurodegenerative pathologies; glycemia may also play a role, by reducing uptake in healthy organs, although this impact has not been significant in published studies.

Further studies are awaited to evaluate more precisely the place of these ratios in relation to the other prognostic indexes and scores already established.
